# Musculoskeletal Injuries and Risk Factors in Spanish CrossFit^®^ Practitioners

**DOI:** 10.3390/healthcare11091346

**Published:** 2023-05-07

**Authors:** Lucas Lastra-Rodríguez, Inés Llamas-Ramos, Vicente Rodríguez-Pérez, Rocío Llamas-Ramos, Ana F. López-Rodríguez

**Affiliations:** 1 Medfis Clinic, Avenida Viveiro 9 Bajo, 15403 Ferrol, Spain; lucas.lastr@gmail.com; 2 Faculty of Nursing and Physiotherapy, Department of Nursing and Physiotherapy, Universidad de Salamanca, C/ Donantes de Sangre s/n, 37007 Salamanca, Spain; inesllamas@usal.es; 3 University Hospital of Salamanca, Paseo de San Vicente, 182, 37007 Salamanca, Spain; 4 Faculty of Nursing and Physiotherapy, Department of Nursing and Physiotherapy, Universidad de León, Campus Universitario, AVDA Astorga, s/n, 24401 León, Spain

**Keywords:** strength training, CrossFit^®^, physical exercise, endurance training, adverse effects

## Abstract

CrossFit^®^ Training is a physical and sports-conditioning system based on constantly varied functional movements performed at high intensity. CrossFit^®^ has been shown to significantly improve general physical performance and body composition. Although there seems to be an association between the practice of CrossFit^®^ and musculoskeletal injuries, the relationship between CrossFit^®^ and injury risks has been poorly studied. The main objective of this study was to establish the relationship between CrossFit^®^ and musculoskeletal injuries. Secondary objectives were the analysis of various risk factors and injury and the comparison of the incidence of CrossFit^®^ injuries to that of other sports. An online questionnaire was distributed to gyms affiliated with CrossFit^®^, Inc. in the Principality of Asturias, Spain in order to carry out a retrospective transversal descriptive study. The frequency of injuries in CrossFit^®^ is similar to most sports. Injuries are often minor and of short duration, with the shoulder being the most affected joint complex.

## 1. Introduction

The training method known as CrossFit^®^ is defined as a physical and sports conditioning system based on constantly varied functional movements performed at high intensity. Workouts involve three main elements: cardiovascular exercises (e.g., running, swimming, and rope skipping), gymnastic-type exercises (push-ups or pull-ups) and powerlifting and weightlifting exercises. The characteristics of CrossFit^®^ foster great adherence [1], which has contributed to achieving more popularity. In 2000, there were only 13 CrossFit^®^ affiliated gyms in the United States, and there are now more than 13,000 CrossFit^®^ affiliated gyms worldwide. In Spain, in 2011, there were six centers, and the number increased up to 661 by 2023 [2].

CrossFit^®^ has multiple benefits, significant improvements in general physical performance and body composition in both adolescents [3,4] and adults. Murawska et al. [5] detected improvements in levels of neurotrophic factor resulting in the brains of CrossFit^®^ participants, which could have implications in treatment of eating disorders or depression. CrossFit^®^ might also be useful in the treatment of cardiorespiratory diseases [6], though such therapeutic benefits are still unclear. Although there seems to be an association between the practice of CrossFit^®^ and musculoskeletal injuries, only four epidemiological studies appear in the PubMed database when searching for “CrossFit^®^ injury” [7,8,9,10]. The current scientific literature related to CrossFit^®^ has few studies with a high level of evidence and low risk of bias [11]. Therefore, the relationship between CrossFit^®^ and injuries derived from it should be determined prior to establishing guidelines or protocols for injury prevention.

The main objective of this study is to establish the incidence of musculoskeletal injuries among CrossFit^®^ practitioners. Secondary objectives are the analysis of various risk factors and the comparison of the incidence of injuries between CrossFit^®^ and other sports.

## 2. Materials and Methods

### 2.1. Study Design

An online questionnaire was distributed to gyms affiliated with CrossFit^®^, Inc. in the Principality of Asturias, Spain in order to carry out a retrospective transversal descriptive study. The questionnaire was distributed online during two periods of 2 months.

The present study was carried out in accordance with the Declaration of Helsinki [12].

### 2.2. Study Configuration, Participants, and Sample Size

#### Study Setting and Participants

The sample was composed by people who practiced CrossFit^®^ in the Principality of Asturias. To be considered CrossFit^®^ practitioners they had been training this sport for at least 2 months.

The sample size was determined based on Cohen’s statistical power using the chi-square test [13]. This process was carried out using R software (v 4.3.2., www.r-project.org, accessed on 21 October 2022 ); it was determined that for a statistical power of 80%, it was necessary to have at least 88 subjects.

Inclusion criteria were people who were older than or equal to 16 years and practice CrossFit^®^ in any gym affiliated with CrossFit^®^ Inc., training in the Principality of Asturias and. Training was always guided under a CrossFit^®^ trainer supervision.

### 2.3. Independent Variables

#### 2.3.1. Characteristics of the CrossFit^®^ Practitioner

Data were collected on previous experience in sports, including sports activities, time practicing CrossFit^®^ and numbers of practice hours per week.

#### 2.3.2. Characteristics of Sports Practice

Loads were determined using percentage of the one-repetition maximum (1 RM) and were collected for regular chest, shoulder, leg and lumbar exercises (military press, bench press, squat and deadlift).

#### 2.3.3. Injuries

The questionnaire used in this study included the number of injuries suffered during the past year, details and specific characteristics of the injury. Injuries were classified as minor if they do not affect daily activities (DA) or the practice of CrossFit^®^, medium if they affect DA or severe if they affect both.

### 2.4. Dependent Variables

Relationships between Injuries and Characteristics of the CrossFit^®^ Practitioner (Sex, Weight and Experience in CrossFit® Performing Mobility Exercises during the CrossFit^®^ Session) have been considered as dependent variables.

Relationships between Injuries and Characteristics of Sports Practice, Loads in Strength Exercises and Number of Hours of Practice have been considered as independent variables.

### 2.5. Procedure

For this study “The Incidence of injuries in CrossFit^®^ athletes Questionnaire” was designed. It was made following consultation with five researchers specialized in physical condition and injuries in the locomotor system. The questionnaire was divided into two batteries, following their guidelines: physical and training characteristics of the participants and description of injuries. Before completing the questionnaire, each participant was informed of the objectives of the study and gave informed consent.

Approximate time of completion of the questionnaire was 15–20 min, depending on the presence and number of injuries of the participant.

Participants took part voluntarily and did not receive any reward for their participation.

### 2.6. Statistical Considerations

IBM SPSS Statistics 27.0 software for Windows was used to analyze all of the descriptive variables addressed in the study.

The chi-square test was used to establish the existence of a relation between variables at a confidence level of 95%.

## 3. Results

### 3.1. Characteristics of the CrossFit^®^ Practitioner

**Figure 1:**
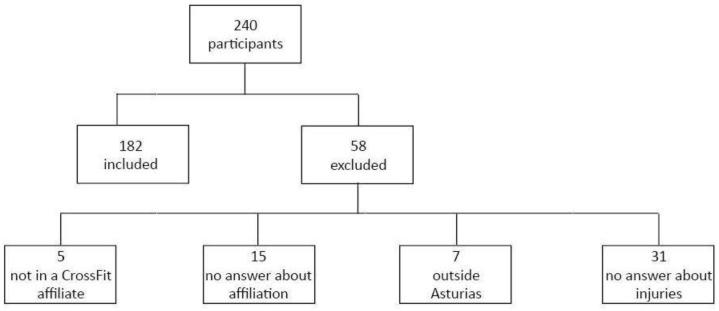
Sampling.

In total, 240 practitioners answered the survey. After the inclusion and exclusion criteria were applied, 58 people were excluded because their gym was not affiliated with CrossFit^®^, they were outside the selected region or did not answer questions about injuries. Finally, 182 participants were included in the present study, 118 men (65%) and 64 women (35%) who practiced CrossFit^®^ in the Principality of Asturias (Figure 1).

In the descriptive analysis, the majority of the respondents (83%) practiced any sport for at least one year. Beginners were considered as subjects with 1 year or less of practice (*n* = 64) and advanced as those with more than 1 year of practice (*n* = 118).

In total, 95.7% of the participants had previous experience in any sports or physical activity, and for 8.8% of them, CrossFit^®^ was their first experience in the sports field.

**Figure 2:**
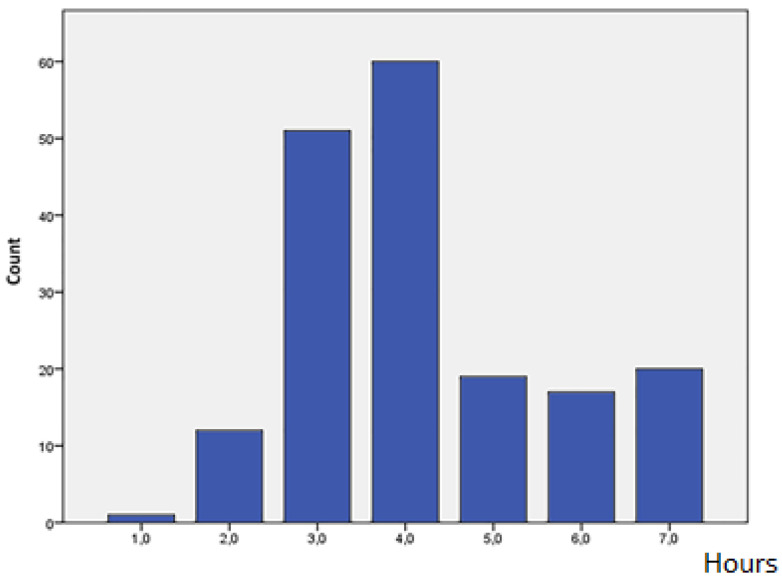
Weekly hours of CrossFit^®^ practice.

Most participants (61%) practice sports 3 or 4 h per week (hpw) and the lowest frequency group practice them 1 or 2 h (hpw), (7.1%), (Figure 2).

### 3.2. Characteristics of Sports Practice

While the majority of the participants were beginners in CrossFit^®^, the physical condition in terms of muscular strength was considered good.

In this study, 1 RM was analyzed in four basic exercises at CrossFit^®^: military press, bench press, squat and deadlift. 

The levels of strength of 1.25 kg per bodyweight (BW) in the bench press, 0.75 kg per BW in the military press and 1.8 kg per BW in the squat and deadlift were used to categorize participants as novice-intermediate lifters [14].

The most frequent levels of strength collected were 75% of their body weight in military press (n = 82) and bench press (n = 60) and 150% in squats (n = 61) and deadlifts (n = 68).

### 3.3. Incidence of Injuries

A total of 143 injuries were recorded among the 182 respondents, distributed as follows (Table 1): absence of injuries, 79 participants (43.3%); one injury, 69 participants (37.91%); two injuries, 29 participants (15.9%); three injuries, four participants (2.2%) and four injuries, one participant (0.5%).

The rate of injuries per hour of CrossFit^®^ was established as 3.6/1000 h. Data on first injuries showed that in most cases injuries were of medium severity, without affecting daily life activities of the respondent. As for duration of injury, 28% (n = 18) lasted 1 month, 20% lasted 2 months (n = 20) and 17% lasted 2 weeks (n = 8).

The severity of second injuries was greater than that of first injuries. Duration of injury was shorter, either 1 month (n = 10) or 1 week (n = 8).

Data on the third and fourth injuries provided little information due to the small number of cases (n = 5).

#### 3.3.1. Location of Injuries

**Figure 3:**
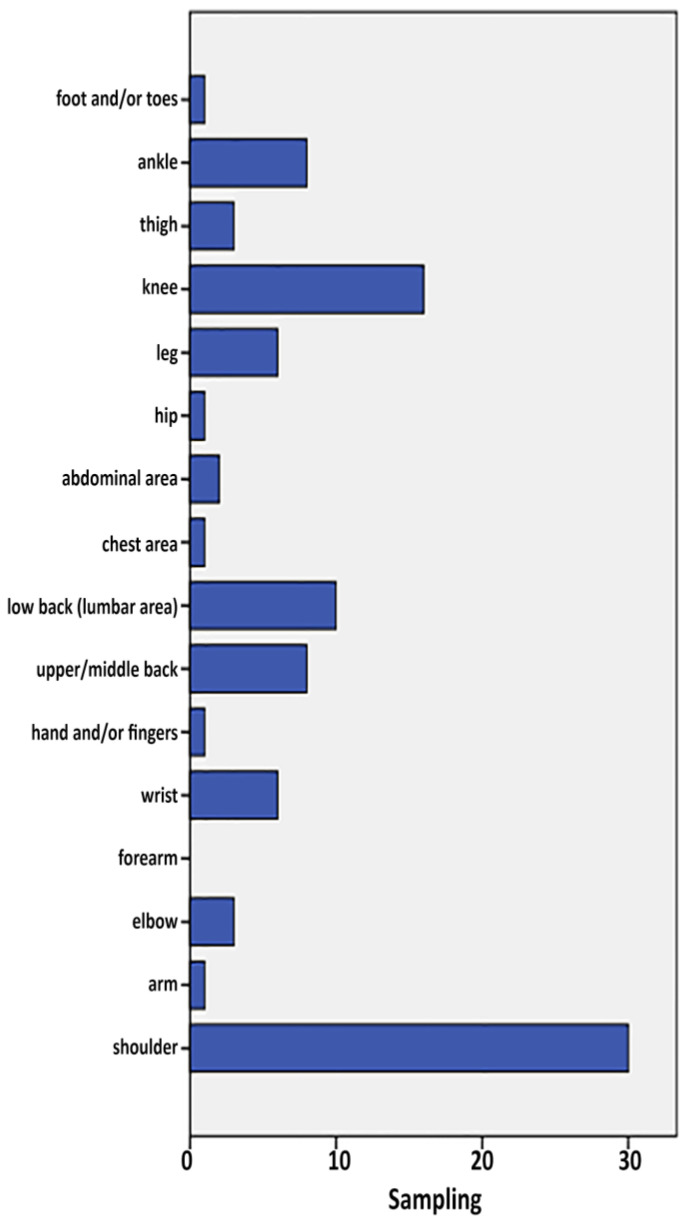
Injury area of first injury.

First injuries occurred mostly in the shoulder (29.1%, n = 30), back (17.5%) and knee (15.5%) (Figure 3). The distribution is more equitable among second injuries, with the most common being in the upper back (7.5%, n = 4), although this is not significant due to the small number of respondents with two injuries (n = 17).

#### 3.3.2. Injury Mechanism and Relapse

Overuse or overtraining was the most common factor for first (50%) and second injuries (44%). First injury was not recurrent in 70% of the cases (n = 70), while among those with second injuries, there was a very similar distribution between non-recurring (16%) and recurring injuries (12%).

#### 3.3.3. Mobility

Mobility exercises before CrossFit^®^ sessions are very important. In order to determine whether the frequency of work on mobility is related to the presence of injury, questions on frequency of mobility were included in the questionnaire as follows: never, almost never, almost always and always.

#### 3.3.4. Incidence of Injuries and Hours of Practice

A relationship was shown between mobility and the number of weekly hours of CrossFit^®^ training ( *p* = 0.021; Table 2 and Table 3).

No relationship was found between variables of sex, weight, experience in CrossFit^®^ or strength and the presence of injury.

#### 3.3.5. Treatment of Injuries

Among the respondents with first injury, 24% (n = 24) did not follow treatment.

Second injury seemed to receive more attention, with only 7.8% (n = 8) not applying treatment. When following treatment, physiotherapy was the preferred choice in most cases (n = 77), followed by pharmacological treatment or alternative therapies, such as acupuncture.

Among the sample of 182 respondents, only three injuries ended in surgical operation.

#### 3.3.6. Correlations

Dependencies between sex, weight, experience, the frequency of mobility exercises, weekly volume of training, and general strength were analyzed.

### 3.4. Incidence of Injuries and Frequency of Mobility Exercises

There was a relationship between the number of injuries and the frequency of performing mobility exercises (*p* = 0.01; Table 4 and Table 5).

### 3.5. The Incidence of Injuries and Hours of Practice

There was a correlation between the weekly hours of CrossFit^®^ practice and the presence of injuries (*p* = 0.023; Table 6 and Table 7).

## 4. Discussion

Currently, CrossFit^®^ training is increasing world-wide. An adequate knowledge of the relationship between CrossFit^®^ practice and injury presence is necessary. CrossFit^®^ has showed higher levels of satisfaction and motivation [11]; however, there is little information on therapeutic benefits of CrossFit^®^. Murawska et al. point to an improvement in levels of neurotrophic factor, an important molecule in trophism and neuronal plasticity [5]. CrossFit^®^ training, with the appropriate adaptations, could benefit patients with cardiorespiratory diseases, due to its high-intensity interval training (HIIT) [6].

Currently, CrossFit^®^ practice has increased as has the number of publications related to the characteristics of the associated injuries [15,16,17]. Furthermore, it has been studied all across the world in countries such as Portugal, Brazil and the Netherlands [18,19,20]. To our knowledge, in Spain there is little scientific evidence on these associated injuries; hence, this study has been carried out. 

The high intensity and complexity of some CrossFit^®^ exercises raise safety concerns [21,22]. With a perceived level of effort considerably greater than in other types of training, it is necessary to consider a period of adaptation in CrossFit^®^, individually moderating training intensity and difficulty of the training and adding appropriate progressions and programming phases.

### 4.1. Comparison with Other Sports

Studies of similar characteristics in other sports were used to compare the results obtained in this study, which include activities practiced within CrossFit^®^, such as gymnastic exercises [23] strength disciplines (e.g., weightlifting [24], weight training [25,26], powerlifting [17,27,28], strongman [29] and bodybuilding [30]). Other comparative studies have recorded injury ratios in popular sports, such as running [31,32,33] and track and field [34], as well as in team sports, such as rugby [35], handball, volleyball and floorball [36].

The injury risk from CrossFit^®^ training is comparable to Olympic weightlifting, distance running, track and field, rugby, football, ice hockey, soccer or gymnastics.

Almost all of the above-cited studies focus on high-level athletes and, in most cases, professionals, making it difficult to compare the data collected since the workload of professional athletes cannot be compared to that of amateur practitioners.

#### 4.1.1. Incidence and Prevalence of Injuries

The incidence found in previous studies is mixed; however, it could be established between 0.2 and 18.9/1000 h of exposure [17]. In their review implemented in Brazil, Serafin et al. showed an injury incidence of 3.51/1000 h of exposure [19]. This data agrees with the results of our study 3.6/1000 h of exposure. 

Prevalence of injuries is variable, as reported in previous studies on CrossFit^®^: 19.4% [7], 26% [8] and 73.5% [9]. However, according to other studies, the prevalence of injuries in other training methods or sports varies and is similar to the 56.6% found in the present study of practitioners of CrossFit^®^. In the systematic review by van Gent et al. [33], the prevalence of lower extremity injuries among long-distance runners varies from 19.4% to 79.3%.

Studies of sports with exercises performed within CrossFit^®^ sessions, including powerlifting (a strength sport consisting of three basic lifts, squat, deadlift and bench press) and weightlifting, report that 43.3% of participants suffer from some kind of pain [24,27]. Among powerlifting professionals, 64.2% reported suffering an injury during a 6-year period [30] and strongman athletes were the most frequently injured (82% injured within a 1-year period) [29].

#### 4.1.2. Location of Injuries

In the present study, the most affected body area was the scapulohumeral joint complex. Shoulder involvement is commonly documented in many studies in various sports [19,20,37] mostly with a slightly lower injury rate than the one found here. Keogh et al. [27] and Siewe et al. [28] reported a similar percentage (29.1%); both referring to powerlifting. Similar results are shown in the study led by Weisenthal et al. [7], (25%, n = 21), and Hak et al. [10]. In gymnastics, directly linked to CrossFit^®^, incidence of shoulder injuries ranges from 16.8% to 19% [23]. In other sports, such as handball or rugby, injuries of this joint complex are also within the range 15–30% [35,36]. These data may indicate greater harm to this joint complex compared to other sports; therefore, the reasons for such results would be an interesting field of investigation. 

The back (17.5%) was among the most frequently injured areas of the body, similar data were found in Brazil (18.3%) [19]; however, the prevalence of back injuries was higher in the Netherlands (24%). These problems are usually documented in studies on both non-athletes and athletes in different sports [38]. Some movements usually performed during CrossFit^®^ sessions, such as deadlifts or weightlifting exercises, expose the spine (especially the lower back) to compression situations that might increase the risk of injury [39,40]. The ratios of high, medium or low back injuries vary from 24% to 29% in various sports studies, including powerlifting, strongman or floorball [27,29,36].

The back injury frequency of 17.5% reported in the present study is similar to the results of other studies, and therefore, according to such data, the practice of CrossFit^®^ does not present a higher risk of back injury compared to other sports. Similar results have also been obtained in CrossFit^®^ athletes with a highly variable incidence between 17% and 73.5% [7,8]. 

The knee (15.5%) was also one of the most injured body areas in this study. This area is most affected in high impact sports. Amongst runners, reported knee injury percentages between 7.2% and 50% are higher than the one in the present study [31,33]. Percentages in team sports are higher than in CrossFit^®^, with incidences of 36% in volleyball and 27% in floorball among Norwegian athletes [36]. In a retrospective study carried out in Oceania, in powerlifting [27], results (9%) are similar to the ones recorded in the present study.

Knee injury ranges were mostly lower than those reported in other sports, which might indicate that training CrossFit^®^ has some degree of protection against knee injuries. As indicated by previous studies [41], moves such as squat (performed as back squat and in other forms) was considered harmful but do not result in a higher level of knee injuries.

In 65.1% of the cases, injuries occurred during CrossFit^®^ sessions or competitions. This could be due to the average number of hours per week of CrossFit^®^ practice (4.3 h/wk), which is greater than in other physical activities (2.4 h/wk). Further research should focus on identifying factors that increase the risk of injury, especially those not covered by the present study, such as training sessions scheduled by coaches and frequency of certain exercises.

### 4.2. Methodological Considerations

Although the questionnaire used in this study is not validated, the above mentioned analysis of the power of the study shows that at least 88 subjects are necessary to reject the null hypothesis. Therefore, a study with 182 subjects could be considered as a valid and significant reference.

Data are self-reported, so participants could be biased due to selective memory and overstatement, and should be considered when interpreting the data. Furthermore, CrossFit^®^ is an individualized practice, where the trainers adapt the exercises and the loads to each participant. This issue does not influence our results because the objective of the study was to establish the injury prevalence in comparison with other sports, not which exercise or load causes injuries.

Results with a higher level of veracity and validity are those related to the presence or absence of injury, number of injuries, and the injured areas [42]. When interpreting the data collected we should consider possible bias among the participants impacting the veracity of the data. 

The sample collected was limited because only Spanish regions were considered and this limited the analysis of the different risk factors that can increase the possibility of injury and. In order to establish relationships among the different variables it was necessary to divide results into two different groups, which limited, to a great extent, the reliability of the results. To arrive at more specific conclusions, we would need a much higher sample, and therefore, a higher number of injured subjects. With a greater range of data, we could establish a normal distribution of the sample and perform a k-s study which would give us information on the goodness of fit.

## 5. Conclusions

The injuries reported in this study were frequently minor and of short duration, with the shoulder being the most affected joint complex. Therefore, efforts must focus on detecting dangerous movement patterns in sports practice and include appropriate progressions.

The characteristics of the study sample prevented identification of factors related to risk or injury prevention; thus, future investigations with larger samples would be interesting. The growing popularity of CrossFit^®^ requires greater knowledge of the risks inherent to this discipline. More research is needed in the field of CrossFit^®^ to improve the classification of these injuries and to determine, as proposed in this study, the relationships between injuries and level of the athlete, and whether these injuries are specific to the nature of CrossFit^®^. Another topic that should be tested is the use of additional training material, such as a lumbar belt or knee pads, often used in both CrossFit^®^ and strength sports, in the reduction of the risk of injury.

The inclusion of professional athletes of the highest level in CrossFit^®^ is important in the design of future epidemiological studies. Findings from studies involving professionals could then be compared with those of amateur athletes.

## Figures and Tables

**Table 1:** Injuries incidence during 2015–2016.

		Frequency	Percentage
Valid	0.0	79	43.4
	1.0	69	37.9
	2.0	29	15.9
	3.0	4	2.2
	4.0	1	0.5
	Total	182	100.0

**Table 2:** Mobility and presence of injuries cross tabulation.

			Injuries		Total
			0	1	
Mobility	Frequent	Count	71	79	150
		Expected Count	65.1	84.9	150.0
	Infrequent	Count	8	24	32
		Expected Count	13.9	18.1	32.0
Total		Count	79	103	182
		Expected Count	79.0	103.0	182.0

**Table 3:** Mobility and presence of injury chi-square test.

	Value	gl	Asymptotic Significance (2 Sided)	Exact Significance (2 Sided)	Exact Significance (1 Sided)
Pearson’s chi-square test	5.355 ^a^	1	0.021		
Continuity correction ^b^	4.484	1	0.034		
Likelihood ratio	5.625	1	0.018		
Fisher exact test				0.030	0.016
N of Valid Case	182				

^a^. 0 cells (0.0%) have expected a count smaller than 5. The minimum expected count is 13.89, ^b^. Only calculated for a 2 × 2 table.

**Table 4:** Mobility and number of injuries per year cross tabulation.

			Number of Injuries			Total
			0.0	1.0	2.0	
Mobility	Frequent	Count	71	58	19	148
		Expected Count	66.1	57.7	24.2	148.0
	Infrequent	Count	8	11	10	29
		Expected Count	12.9	11.3	4.8	29.0
Total		Count	79	69	29	177
		Expected Count	79.0	69.0	29.0	177.0

**Table 5:** Mobility and number of injuries chi-square test.

	Value	gl	Asymptotic Significance (2 Sided)
Pearson’s chi-square test	9.202 ^a^	2	0.010
Likelihood ratio	8.173	2	0.017
N of Valid Case	177		

^a^. 1 cells (16.7%) have expected a count smaller than 5. The minimum expected count is 4.75.

**Table 6:** Weekly hours of CrossFit^®^ practice and presence of injuries cross tabulation.

			Injuries		Total
			0	1	
Weekly hours of CrossFit^®^ practice	0–3 h	Count	36	28	64
		Expected Count	27.4	36.6	64.0
	4–5 h	Count	29	50	79
		Expected Count	33.8	45.2	79.0
	≥6 h	Count	12	25	37
		Expected Count	15.8	21.2	37.0
Total		Count	77	103	180
		Expected Count	77.0	103.0	180.0

**Table 7:** Weekly hours of CrossFit^®^ practice and presence of injuries chi-square test.

	Value	gl	Asymptotic Significance (2 Sided)
Pearson’s chi-square test	7.552 ^a^	2	0.023
Likelihood ratio	7.550	2	0.023
Fisher exact test	6.495	1	0.011
N of Valid Case	180		

^a^. 0 cells (0.0%) have expected a count smaller than 5. The minimum expected count is 15.83.

## Data Availability

The data that support the findings of this study are available on reasonable request from the corresponding author.
